# Supplementary material for: Dynamics in typewriting performance reflect mental fatigue during real-life office work
Source: PLoS One. 2020 Oct 6;15(10):e0239984. doi: 10.1371/journal.pone.0239984 (PMC7537853; doi:10.1371/journal.pone.0239984)
Supplement: S1 Appendix — (DOCX) [file pone.0239984.s001.docx]

**S1 Appendix**

**Questionnaire with demographic and work-related questions**

1. Wat is je geboortejaar?
2. Wat is je geslacht?
3. Man
4. Vrouw
5. Hoeveel dagen werk je per week (gemiddeld)?
6. 1
7. 2
8. 3
9. 4
10. 5
11. Hoe ga je naar je werk? Geef het aantal dagen per week per vervoersmiddel aan.

.. dagen met de auto

.. dagen met het openbaar vervoer

.. dagen met de fiets

.. dagen lopend

Anders, namelijk:

1. Hoeveel kilometer reis je naar je werk? (enkele reis)
2. Gebruik je applicaties of producten om je gezondheid of beweeggedrag te monitoren, of heb je dit in het verleden gedaan? Denk bijvoorbeeld aan een Fitbit, Nike Fuelband, Runkeeper of Strava
3. Nee, ik heb nooit apps of producten gebruikt om mijn gezondheid of 
   beweeggedrag te monitoren
4. Ja, ik gebruik nu een app of product, namelijk:
5. Ja, ik heb in het verleden een app of product gebruikt, namelijk:
6. Sport je regelmatig?
7. Ja, ik sport meerdere keren per week
8. Ja, ik sport eens per week
9. Nee, ik sport niet regelmatig
10. Hoe veel uur per week sport je gemiddeld?
11. Rook je?
12. Ja
13. Nee
14. Wat is je functiecategorie binnen de faculteit Economie en Bedrijfskunde?
15. Wetenschappelijk personeel (WP)
16. Ondersteunend en beheerspersoneel (OBP)
17. Hoe zou je je werkplekverdeling omschrijven?
18. Ik werk meestal op kantoor (75­100% kantoortijd)
19. Ik werk regelmatig op kantoor, maar ook soms op andere locaties (50­75% kantoortijd)
20. Ik werk soms op kantoor, maar ook regelmatig op andere locaties (25­50% kantoortijd)
21. Ik werk meestal op andere locaties (0­25% kantoortijd)
22. Hoe zou je je taakgerichtheid of focus tijdens je werk omschrijven?
23. Als ik een taak moet afmaken richt ik mij volledig op die taak en wil ik niet gestoord worden
24. Als ik een taak moet afmaken richt ik mij voornamelijk op die taak, maar kan ik wel gestoord worden voor belangrijke zaken
25. Als ik een taak moet afmaken doe ik dat terwijl ik ook andere belangrijke dingen doe en hiervoor gestoord kan worden
26. Als ik een taak moet afmaken doe ik dat terwijl ik ook andere belangrijke en minder belangrijke dingen doe, en kan ik voor alles gestoord worden
27. Welke gezondheidsgerelateerde hulpmiddelen of programma's van de RUG gebruik je of heb je in het verleden gebruikt? (meerdere antwoorden mogelijk)
28. Geen
29. BALANS­activiteiten
30. de Health Check
31. Een hoog­laagbureau
32. Een aangepaste bureaustoel
33. Een fietsbureaustoel
34. Anders, namelijk:
35. Heb je last of last gehad van werkgerelateerde fysieke klachten? (denk aan: RSI, lage rugpijn)
36. Ja, ik heb nu last van werkgerelateerde fysieke klachten
37. Ja, ik heb in het verleden last gehad van werkgerelateerde fysieke klachten
38. Nee, ik heb geen last (gehad) van werkgerelateerde fysieke klachten
39. Heb je last of last gehad van werkgerelateerde mentale klachten? (denk aan: burnout,

depressie, stress)

1. Ja, ik heb nu last van werkgerelateerde mentale klachten
2. Ja, ik heb in het verleden last gehad van werkgerelateerde mentale klachten
3. Nee, ik heb geen last (gehad) van werkgerelateerde mentale klachten
4. Hoeveel uren geef je per week (gemiddeld) les in blok IIB?
5. Hoeveel uren geef je per week (gemiddeld) les in blok IA volgend jaar (2017­2018)?
6. Heb je plannen om tijdens blok IIB of IA een periode (3 of meer werkdagen) weg te zijn, in verband met bijvoorbeeld vakantie of conferentiebezoek? Zo ja, wanneer?
7. Is de computer op je werkplek beheerd door het CIT of door jezelf?
8. Beheerd door het CIT
9. Beheerd door mezelf
10. Ik weet het niet
11. Wat is je mobiele telefoonnummer? Dit nummer wordt alleen gebruikt voor het versturen van een deel van de feedbackberichten.

**English translation of questionnaire with demographic and work-related questions**

1. What is your year of birth?

2. What is your gender?

a. Man

b. Woman

3. How many days do you work per week (on average)?

a. 1

b. 2

c. 3

d. 4

e. 5

4. How do you go to work? Indicate the number of days per week per means of transport.

.. days by car

.. days by public transport

.. days by bike

.. walking for days

Otherwise, namely:

5. How many kilometers do you travel to work? (one way)

6. Do you use applications or products to monitor your health or exercise behavior, or have you done this in the past? Think for example of a Fitbit, Nike Fuelband, Runkeeper or Strava

a. No, I have never used apps or products to improve my health or monitor exercise behavior

b. Yes, I now use an app or product, namely:

c. Yes, I have used an app or product in the past, namely:

7. Do you exercise regularly?

a. Yes, I exercise several times a week

b. Yes, I exercise once a week

c. No, I don't exercise regularly

8. How many hours a week do you exercise on average?

9. Do you smoke?

a. Yes

b. No

10. What is your job category within the Faculty of Economics and Business?

a. Scientific staff (WP)

b. Support and management staff (OBP)

11. How would you describe your workplace distribution?

a. I usually work in the office (75-100% office time)

b. I regularly work in the office, but also sometimes in other locations (50-75%

office time)

c. I sometimes work in the office, but also regularly in other locations (25-50%

office time)

d. I usually work in other locations (0-25% office time)

12. How would you describe your task orientation or focus during your work?

a. If I have to finish a task, I focus entirely on that task and I do not want to

to be interrupted

b. When I have to finish a task, I mainly focus on that task, but I can

disturbed for important matters

c. If I have to finish a task, I do it while doing other important things

do and can be disturbed for this

d. If I have to finish a task, I do it while I also other important and

do minor things, and can I be disturbed for anything

13. What health-related aids or programs at the RUG do you use or have you used in the past? (multiple answers possible)

a. None

b. BALANCE SHEET activities

c. the Health Check

d. A high-low desk

e. A custom office chair

f. A bicycle office chair

g. Otherwise, namely:

14. Have you been bothered or troubled by work-related physical complaints? (think: RSI, low back pain)

a. Yes, I now suffer from work-related physical complaints

b. Yes, I have had work-related physical complaints in the past

c. No, I do not (have) suffered from work-related physical complaints

15. Have you been bothered or troubled by work-related mental complaints? (think: burnout,

depression, stress)

a. Yes, I now suffer from work-related mental complaints

b. Yes, I have had work-related mental complaints in the past

c. No, I do not (have) suffered from work-related mental complaints

16. How many hours per week (on average) do you teach in block IIB?

17. How many hours per week (on average) do you teach in block IA next year (2017-2018)?

18. Do you have plans to be away for a period (3 or more working days) during block IIB or IA, for example due to holidays or conference attendance? If so, when?

19. Is the computer at your workplace managed by the CIT or by yourself?

a. Managed by the CIT

b. Managed by myself

c. I do not know

20. What is your mobile phone number? This number is only used for sending part of the feedback messages.
