# Supplementary material for: Dynamics in typewriting performance reflect mental fatigue during real-life office work
Source: PLoS One. 2020 Oct 6;15(10):e0239984. doi: 10.1371/journal.pone.0239984 (PMC7537853; doi:10.1371/journal.pone.0239984)
Supplement: S2 Appendix — (DOCX) [file pone.0239984.s002.docx]

**S2 Appendix**

**Weekly questionnaire**

1. We zijn benieuwd of je wat hebt gehad aan de feedback die je de afgelopen week per mail en SMS hebt ontvangen. Geef aan in welke mate je het eens bent met onderstaande uitspraken.
2. Ik heb de feedback in de afgelopen week gelezen - *Volledig mee eens, Deels mee eens, Niet mee eens/niet mee oneens, Deels mee oneens, Volledig mee oneens.*
3. Ik vind de feedback van de afgelopen week betrouwbaar - *Volledig mee eens, Deels mee eens, Niet mee eens/niet mee oneens, Deels mee oneens, Volledig mee oneens.*
4. De constateringen m.b.t. mijn gedrag en omgeving kwamen overeen met mijn eigen inschatting - *Volledig mee eens, Deels mee eens, Niet mee eens/niet mee oneens, Deels mee oneens, Volledig mee oneens.*
5. De tweede set vragen gaat over de mate waarin je plant of bedenkt hoe je je gedrag verandert. Dus: "Als het gaat om mijn dagelijkse gezondheid en beweeggedrag..."
6. ga ik met de data van de sensoren planmatig te werk - *Volledig mee eens, Deels mee eens, Niet mee eens/niet mee oneens, Deels mee oneens, Volledig mee oneens.*
7. stel ik mezelf doelen met betrekking tot de data van de sensoren - *Volledig mee eens, Deels mee eens, Niet mee eens/niet mee oneens, Deels mee oneens, Volledig mee oneens.*
8. besteed ik aandacht aan het plannen van activiteiten aan de hand van de data van de sensoren - *Volledig mee eens, Deels mee eens, Niet mee eens/niet mee oneens, Deels mee oneens, Volledig mee oneens.*
9. De derde set vragen gaat over de mate waarin je je geplande verandering uitvoert. Dus: "Als het gaat om mijn dagelijkse gezondheid en beweeggedrag..."
10. komt het vaak voor dat ik dingen anders doe dan ik met mezelf door gebruik van de sensoren heb afgesproken - *Volledig mee eens, Deels mee eens, Niet mee eens/niet mee oneens, Deels mee oneens, Volledig mee oneens.*
11. komt het vaak voor dat ik dingen anders doe dan ik met mezelf door gebruik van de sensoren heb afgesproken - *Volledig mee eens, Deels mee eens, Niet mee eens/niet mee oneens, Deels mee oneens, Volledig mee oneens.*
12. komt wat ik bedenk door gebruik van de sensoren en wat ik doe overeen - *Volledig mee eens, Deels mee eens, Niet mee eens/niet mee oneens, Deels mee oneens, Volledig mee oneens.*
13. Hoeveel uren verwacht je werkgever dat je in een normale 7-daagse werkweek werkt? (Als dit varieert, geef een schatting van het gemiddelde)
14. Hoeveel uren heb je in de afgelopen 7 dagen in totaal ongeveer gewerkt?
15. Geef hieronder aan hoeveel procent van deze werkuren van de afgelopen 7 dagen je op je eigen kantoor, thuis, of elders hebt gewerkt. Het totaal moet 100 zijn.

Op mijn eigen kantoor : _______ (1)

Thuis : _______ (2)

Elders : _______ (3)

Totaal : ________

1. Op een schaal van 0 tot 10, waarbij de score 0 overeenkomt met de slechtst mogelijke prestatie en de score 10 met de best mogelijke prestatie in uw werk, hoe beoordeel je de werkprestatie van de meeste werknemers die vergelijkbaar werk doen?
2. Hoe zou je op dezelfde schaal van 0 tot 10 je algehele werkprestatie beoordelen in de afgelopen 7 dagen?

**English translation of weekly questionnaire**

1. We are curious if you have benefited from the feedback you have received by email and SMS in the past week. Please indicate to what extent you agree with the statements below.
2. I have read the feedback in the past week - *I completely agree, I partly agree, I don’t agree / I don’t disagree, I partly disagree, I completely disagree.*
3. I find the feedback from the past week reliable - *I completely agree, I partly agree, I don’t agree / I don’t disagree, I partly disagree, I completely disagree.*
4. The observations regarding my behavior and environment corresponded to my own assessment - *I completely agree, I partly agree, I don’t agree / I don’t disagree, I partly disagree, I completely disagree.*
5. The second set of questions is about the degree to which you plan or consider how to change your behavior. So: "When it comes to my daily health and exercise behavior ..."
   1. I proceed systematically with the data from the sensors - *I completely agree, I partly agree, I don’t agree / I don’t disagree, I partly disagree, I completely disagree.*
   2. I set goals with regard to the sensor data - *I completely agree, I partly agree, I don’t agree / I don’t disagree, I partly disagree, I completely disagree.*
   3. I pay attention to planning activities based on the sensor data - *I completely agree, I partly agree, I don’t agree / I don’t disagree, I partly disagree, I completely disagree.*
6. The third set of questions is about the extent to which you are making your planned change. So: "When it comes to my daily health and exercise behavior ..."
7. it often happens that I do things differently than I have agreed with myself by using the sensors *- I completely agree, I partly agree, I don’t agree / I don’t disagree, I partly disagree, I completely disagree.*
8. It often happens that I do things differently than I agreed with myself using the sensors - *I completely agree, I partly agree, I don’t agree / I don’t disagree, I partly disagree, I completely disagree.*
9. what I think of using the sensors and what I do matches - *I completely agree, I partly agree, I don’t agree / I don’t disagree, I partly disagree, I completely disagree.*
10. How many hours does your employer expect you to work in a normal 7-day work week? (If this varies, please estimate the average)
11. In total, how many hours did you work in the past 7 days?
12. Please indicate below what percentage of these working hours of the past 7 days you have worked in your own office, at home or elsewhere. The total must be 100.

At my own office: _______ (1)

At home: _______ (2)

Elsewhere: _______ (3)

Total: ________

1. On a scale from 0 to 10, where the score 0 corresponds to the worst possible performance and the score 10 the best possible performance in your work, how do you rate the work performance of most employees who do similar work?
2. How would you rate your overall job performance on the same scale from 0 to 10 in the past 7 days?
